# Identification of the Essential *Brucella melitensis* Porin Omp2b as a Suppressor of Bax-Induced Cell Death in Yeast in a Genome-Wide Screening

**DOI:** 10.1371/journal.pone.0013274

**Published:** 2010-10-11

**Authors:** Géraldine Laloux, Michaël Deghelt, Marie de Barsy, Jean-Jacques Letesson, Xavier De Bolle

**Affiliations:** Research Unit in Molecular Biology, Department of Biology, University of Namur (FUNDP), Namur, Belgium; Universidad Nacional, Costa Rica

## Abstract

**Background:**

Inhibition of apoptosis is one of the mechanisms selected by numerous intracellular pathogenic bacteria to control their host cell. *Brucellae*, which are the causative agent of a worldwide zoonosis, prevent apoptosis of infected cells, probably to support survival of their replication niche.

**Methodology/Principal Findings:**

In order to identify *Brucella melitensis* anti-apoptotic effector candidates, we performed a genome-wide functional screening in yeast. The *B. melitensis* ORFeome was screened to identify inhibitors of Bax-induced cell death in *S. cerevisiae*. *B. melitensis* porin Omp2b, here shown to be essential, prevents Bax lethal effect in yeast, unlike its close paralog Omp2a. Our results based on Omp2b size variants characterization suggest that signal peptide processing is required for Omp2b effect in yeast.

**Conclusion/Significance:**

We report here the first application to a bacterial genome-wide library of coding sequences of this “yeast-rescue” screening strategy, previously used to highlight several new apoptosis regulators. Our work provides *B. melitensis* proteins that are candidates for an anti-apoptotic function, and can be tested in mammalian cells in the future. Hypotheses on possible molecular mechanisms of Bax inhibition by the *B. melitensis* porin Omp2b are discussed.

## Introduction

During the complex evolution of intracellular pathogenic bacteria with their host, mechanisms have been selected that allow bacteria to modulate host cell functions to their advantage. Examples of hijacking of the host cell cytoskeleton [1], signaling [2] and vesicular trafficking [3] by bacterial pathogens are numerous. Pirated processes include apoptosis, a form of programmed cell death that can serve as a defense mechanism to avoid pathogen propagation [4]. Inhibition of apoptosis, which has been reported for a variety of intracellular bacteria, probably supports survival of their replication niche [5].

Monocytes or macrophages infected by the facultative intracellular pathogenic bacteria of the *Brucella* genus, which are responsible for a worldwide zoonosis [6], are protected from apoptosis [7]. Furthermore, modulation of the expression of genes encoding apoptotic proteins in infected cells has been described [7], [8]. The gene encoding an anti-apoptotic protein of the Bcl-2 family, A1/Bfl1, is overexpressed in *Brucella*-infected monocytes compared with non-infected cells [7]. However, the bacterial molecular mechanisms leading to apoptosis inhibition, probably involving production of effector protein(s), are still unknown.

Interestingly, the genetically tractable and easy to handle yeast *Saccharomyces cerevisiae* is known to be an attractive model for the functional study of proteins involved in apoptosis, such as the Bcl-2 family members [9]. The budding yeast lacks homologs of the mammalian core apoptotic proteins, providing a simplified model for individual characterization of apoptosis regulators without the bias due to the rest of the network. Several proteins involved in apoptosis have an effect in yeast that is relevant to their physiological function [9]. For instance, the ectopic production of the mammalian pro-apoptotic Bax in yeast induces a form of cell death, which requires Bax insertion into the outer membrane of mitochondria (OMM). In mammalian cells, Bax translocation to mitochondria occurs upon pro-apoptotic signaling and leads to mitochondrial outer membrane permeabilization (MOMP) [10], a required event for subsequent apoptosis. Pleiotropic effects are observed upon Bax production in yeast, including several features shared with mammalian apoptosis such as MOMP, cytochrome *c* release and the maintenance of plasma membrane integrity [11]. These characteristics, in addition to the fact that Bax-induced cell death in yeast can be reverted by known anti-apoptotic proteins, such as Bcl-2 and Bcl-XL [12], strongly support a common ancestral pathway to controlled cell death. Hence, *S. cerevisiae* has been used as a tool for the identification of new putative apoptosis inhibitors. A screening strategy to identify inhibitors of Bax-induced cell death in yeast [13] has been applied previously to identify new human anti-apoptotic proteins [14], [15], [16] and plant inhibitors of cell death [17], [18]. In addition, assay of Bax-induced cell death inhibition in yeast has contributed to the characterization of human [16] and plant [19] proteins known to be involved in inhibition of apoptotic or hypersensitive response (HR) programmed cell deaths, respectively, as well as bacterial proteins playing a role in host HR cell death inhibition [20] or described as secreted anti-apoptotic effector [21].

Several bacterial effectors that target eukaryotic signaling pathways have been highlighted using diverse functional screening approaches in yeast [22]. The availability of the *Brucella melitensis* ORFeome prompted us to perform a genome-wide functional yeast-based screening to identify *B. melitensis* anti-apoptotic effector candidates. Here we report the results of a screening for Bax suppressors in yeast, applied for the first time to a bacterial genome-wide library of coding sequences.

## Results and Discussion

**Figure 1:**
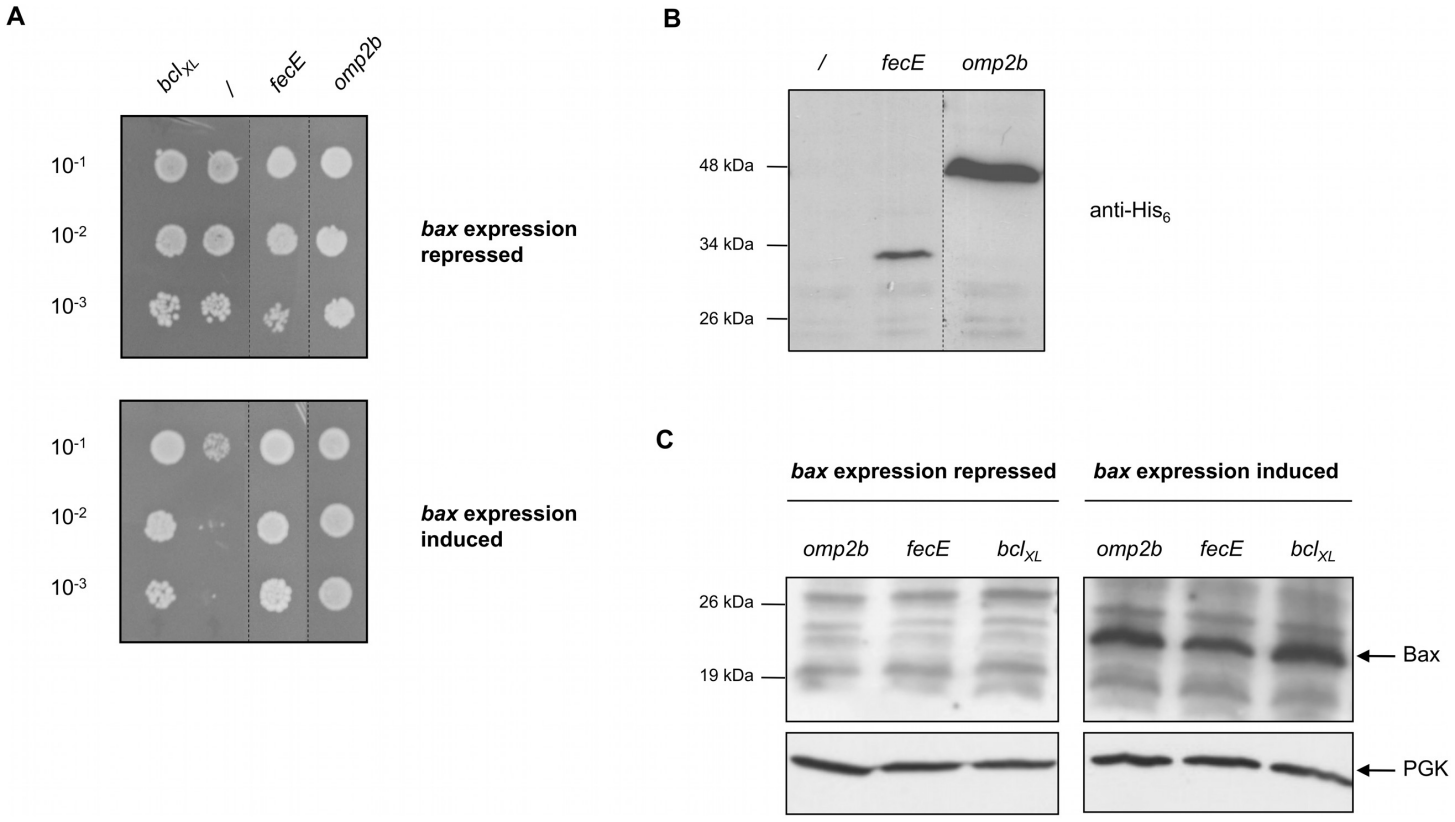
Omp2b and FecE allow yeast growth under *bax* expression conditions. **A.** Drops of serial dilutions of cultures in stationary phase, on media allowing repression or induction of *bax* and ORF expression. Dilution factors are indicated. Pictures were taken after 4 days of growth at 30°C. GL0001 (*bcl_XL_*) and GL0016 (*/*): positive and negative controls for growth, respectively. Growing strains were submitted to PCR and sequencing, identifying *B. melitensis omp2b* (GL0004) and *fecE* (GL0005). **B.** Western blot detection of Omp2b and FecE (fused to a C-terminus His_6_ tag when expressed from pYES-DEST52 vector used for screening), in total protein extracts from GL0002 (*omp2b*) and GL0003 (*fecE*) yeast strains cultured under conditions of *bax* and ORF expression induction. **C.** Western blot detection of Bax (21 kDa) in total protein extracts from GL0004 (*omp2b*), GL0005 (*fecE*) and GL0001 (*bcl_XL_*) yeast strains cultured under conditions of *bax* and ORF expression repression (left part) or induction (right part). Phosphoglycerate kinase (PGK) detection: loading control.

In order to identify *Brucella melitensis* proteins that inhibit Bax-induced cell death in yeast, we took advantage of the *B. melitensis* ORFeome [23], which is a standardized library of predicted coding sequences (ORFs), to construct a yeast library in which each clone contains murine *bax* and one of the ∼3,200 *B. melitensis* ORFs. *bax* and the *B. melitensis* ORFs expression are controlled by the galactose-inducible, glucose-repressible p*GAL10* and p*GAL1* promoters, respectively. This “*bax* + ORFeome” library was screened for colonies with a plasmid-borne growth phenotype under *bax* and ORFs expression condition (see Text S1 in the Supporting information for library construction and further screening details). 116 different ORFs were identified from the 136 colonies obtained at this step of the screen. In order to get rid of false positives that are expected to be selected in this type of large-scale screen of a genome-wide pool of ORFs, the next step of the screening procedure consisted in individually testing all candidates in conventional growth assays [13]. Thus, the 116 pYES-DEST52 ORFs were individually recloned from the original entry vectors of the ORFeome before transformation of a yeast strain containing *bax* (QX95001, see Table 1 for all strains used in this study). Each of the 116 strains was submitted to growth assays (streaks and drops of serial dilutions on plate) under induction or repression conditions of *bax* and ORFs expression. The positive control of growth was GL0001 yeast strain containing *bax* and *bcl_XL_*. Indeed, the anti-apoptotic Bcl-XL protein has been shown to inhibit Bax-induced cell death in yeast [12] as in mammalian cells by preventing Bax insertion into mitochondria and its acquisition of a final conformation able to induce MOMP [24]. The negative control was the GL0016 yeast strain containing *bax* and the empty pYES-DEST52 destination vector. Out of the 116 clones tested, only two yeast strains, expressing *bax* and either *B. melitensis* ORF BMEI1305 or BMEI0660, reproducibly grew as well as the control GL0001 strain (Figure 1A). These ORFs encode Omp2b and FecE proteins, respectively. FecE is predicted to be a cytoplasmic ATP-binding protein associated with other components of an iron (III) ABC transporter. Omp2b is described as a porin [25]. The production of both Omp2b and FecE in yeast (GL0002 and GL0003 strains, respectively) was checked by Western blot analysis (Figure 1B). The growth phenotype conferred by Omp2b or FecE is not linked to an absence or decrease of Bax production as Bax was detected in yeast expressing *bax* and *omp2b* or *fecE* (GL0004 and GL0005, respectively) like in GL0001 strain (Figure 1C). Thus, our screen identified two *B. melitensis* proteins, Omp2b and FecE, of which the expression in yeast prevents cell death induced by the mammalian pro-apoptotic protein Bax. FecE mechanism of action regarding the inhibition of Bax-induced yeast lethality remains to be investigated but may be related to its ATPase activity, considering the various implications of ATPases in intracellular signaling pathways. However we cannot exclude that FecE effect in yeast is independent of this predicted function. Nevertheless, the ability of FecE to act as an anti-apoptotic effector should be tested in the future in other models than the yeast-based system used here as a screening tool.

**Table 1:** Yeast and bacterial strains used in this study.

Name	Strain	Use and relevant features	Medium	Reference and sources
DH10B	*E. coli*; *F^−^ mcrA Δ*(*mrr-hsdRMS-mcrBC*) *phi*80*lacZΔM15 lacX74 recA1 endA1 ara*Δ*139* Δ(*ara leu*)*7697 galU galK lambda^−^ rps* (Str^r^) *nupG*	cloning strain	LB	Gibco BRL
S17-1	*E. coli*; *recA thi pro hsdR* [res– mod+][RP4::2-Tc::Mu-Km::Tn7] lambda-pir phage lysogen	plasmid mobilization in *B. melitensis*	LB	[41]
BF264-15 Dau	*S. cerevisiae* (*MATalpha ade1 his2 leu2-3, 112 trp1-1a ura3*)	yeast strain used in this study for transformation with plasmids harboring *LEU2* or *URA3*	Sc [42]	[43]; kindly provided by M. Grant, Wye College, UK
QX95001	BF264-15 Dau + pYEp51-*bax* [44]	*bax* controlled by p*GAL10* (p*GAL10-bax*)	Sc-Leu	[13]
GL0001	QX95001 + pYES-DEST52-*bcl_XL_*	p*GAL10-bax* + p*GAL1*-*bcl_XL_*-V5-His6	Sc-Leu-Ura	This study
GL0002	BF264-15 Dau + pYES-DEST52-BMEI1305	p*GAL1*-*omp2b*-V5-His6	Sc-Ura	This study
GL0003	BF264-15 Dau + pYES-DEST52-BMEI0660	p*GAL1*-*fecE*-V5-His6	Sc-Ura	This study
GL0004	QX95001 + pYES-DEST52-BMEI1305	p*GAL10-bax* + p*GAL1*-*omp2b*-V5-His6	Sc-Leu-Ura	This study
GL0005	QX95001 + pYES-DEST52-BMEI0660	p*GAL10-bax* + p*GAL1*-*fecE*-V5-His6	Sc-Leu-Ura	This study
GL0007	QX95001 + pYES-DEST52-BMEI1306	p*GAL10-bax* + p*GAL1*-*omp2a*-V5-His6	Sc-Leu-Ura	This study
GL0008	QX95001 + pYES-DEST52-BMEI1249	p*GAL10-bax* + p*GAL1*-*omp25*-V5-His6	Sc-Leu-Ura	This study
GL0010	BF264-15 Dau + pYES-DEST52-*t-omp2b*	p*GAL1*-*t-omp2b*-V5-His6	Sc-Ura	This study
GL0011	QX95001 + pYES-DEST52-*t-omp2b*	p*GAL10-bax* + p*GAL1*-*t-omp2b*-V5-His6	Sc-Leu-Ura	This study
GL0012	BF264-15 Dau + pYES-DEST52- *short-omp2b*	p*GAL1*-*short-omp2b*-V5-His6	Sc-Ura	This study
GL0013	QX95001 + pYES-DEST52-*short-omp2b*	p*GAL10-bax* + p*GAL1*-*short-omp2b*-V5-His6	Sc-Leu-Ura	This study
GL0014	BF264-15 Dau + pYES-DEST52-BMEI1306	p*GAL1*-*omp2a*-V5-His6	Sc-Ura	This study
GL0015	BF264-15 Dau + pYES-DEST52-BMEI1249	p*GAL1*-*omp25*-V5-His6	Sc-Ura	This study
GL0016	QX95001 + pYES-DEST52	p*GAL10-bax* + empty destination vector	Sc-Leu-Ura	This study
GL0017	QX95001 + pYES-DEST52-*long-omp2a*	p*GAL10-bax* + p*GAL1*-*long-omp2a*-V5-His6	Sc-Leu-Ura	This study
GL0018	BF264-15 Dau + pYES-DEST52-*long-omp2a*	p*GAL1-long-omp2a*-V5-His6	Sc-Ura	This study

**Figure 2:**
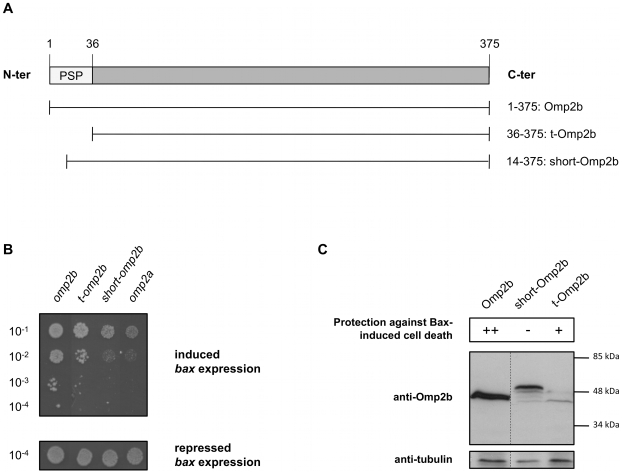
Omp2b signal peptide is required for the Bax-suppressor effect and Omp2b is cleaved in yeast. **A.** Schematic representations of the peptidic sequences of Omp2b variants. Predicted signal peptide (PSP) is represented by a white rectangle in Omp2b sequence. Amino acids positions are indicated. Relative lengths of schematic sequences are on scale. **B.** Growth assay (drops of serial dilutions) on media allowing *bax* and ORF expression repression or induction, for GL0007 (*omp2a*; negative control), GL0004 (*omp2b*; positive control), GL0013 (*short-omp2b*), and GL0011 (*t-omp2b*). Dilution factors are indicated. **C.** Upper part: summary of the growth assays under *bax* and ORF expression induction conditions; “++”: growth, “+”: intermediate growth, “-”: no growth. Lower part: Western blot detection of Omp2b variants, in total protein extracts from GL0002 (Omp2b), GL0010 (t-Omp2b) and GL0012 (short-Omp2b) cultured overnight under expression induction conditions. Tubulin detection: loading control.

Because bacterial porins were already reported to be involved in apoptosis modulation [26], [27], [28], this study focuses on the second candidate, Omp2b. Outer membrane proteins (OMPs) harbor a N-terminal signal peptide (SP) for the recognition and cleavage of the unfolded proteins by the Sec system before their secretion in the periplasm and the folding and insertion of the mature proteins into the bacterial outer membrane [29]. In the *B. melitensis* ORFeome, the deduced sequence from *omp2b* ORF contains a long predicted SP (PSP). A truncated form of Omp2b lacking the entire PSP (t-Omp2b) only poorly inhibits Bax effects in yeast (GL0011) when compared with negative and positive control strains GL0007 and GL0004 (Figure 2A and 2B). This lower protection against Bax lethal effects is not related to a toxicity induced by t-Omp2b since a strain lacking *bax* and producing t-Omp2b (GL0010) grows similarly as the GL0004 strain that produces Omp2b (data not shown). In addition, a variant of Omp2b harboring a N-terminally shortened SP (short-Omp2b) completely looses the anti-Bax effect (GL0013), indicating that the production of Omp2b with its complete PSP is required for a strong Bax inhibition in yeast (Figure 2A and 2B).

Interestingly, Omp2b and t-Omp2b are detected on Western blot at a similar apparent molecular weight, which is lower than the one observed for short-Omp2b (Figure 2C), suggesting that Omp2b PSP is cleaved in yeast, unlike the shortened PSP of the short-Omp2b form. Notably, t-Omp2b was reproducibly detected in a much lesser quantity in Western blot when compared with Omp2b (Figure 2C), which could account for its weaker “anti-Bax” effect. This might suggest that the PSP, and/or the PSP cleavage event, are required for Omp2b production or stability leading to protein levels that are sufficient to remarkably inhibit Bax lethal effects. Altogether, our results support the requirement of PSP cleavage for Omp2b anti-Bax effect in yeast. The processing of Omp2b PSP in yeast is not surprising considering previous studies showing the functionality of bacterial SP in eukaryotic cells [30], [31], [32]. We hypothesize that the processed mature form of Omp2b could adopt a fold allowing its particular subcellular localization or interaction with physical partners, which might lead to the inhibition of Bax-induced effects. This is supported by the Western blot detection of a high molecular weight form of Omp2b in a GL0002 strain protein extract, in semi-denaturing conditions, at a size expected for the trimeric assembly of the porin (data not shown).

**Figure 3:**
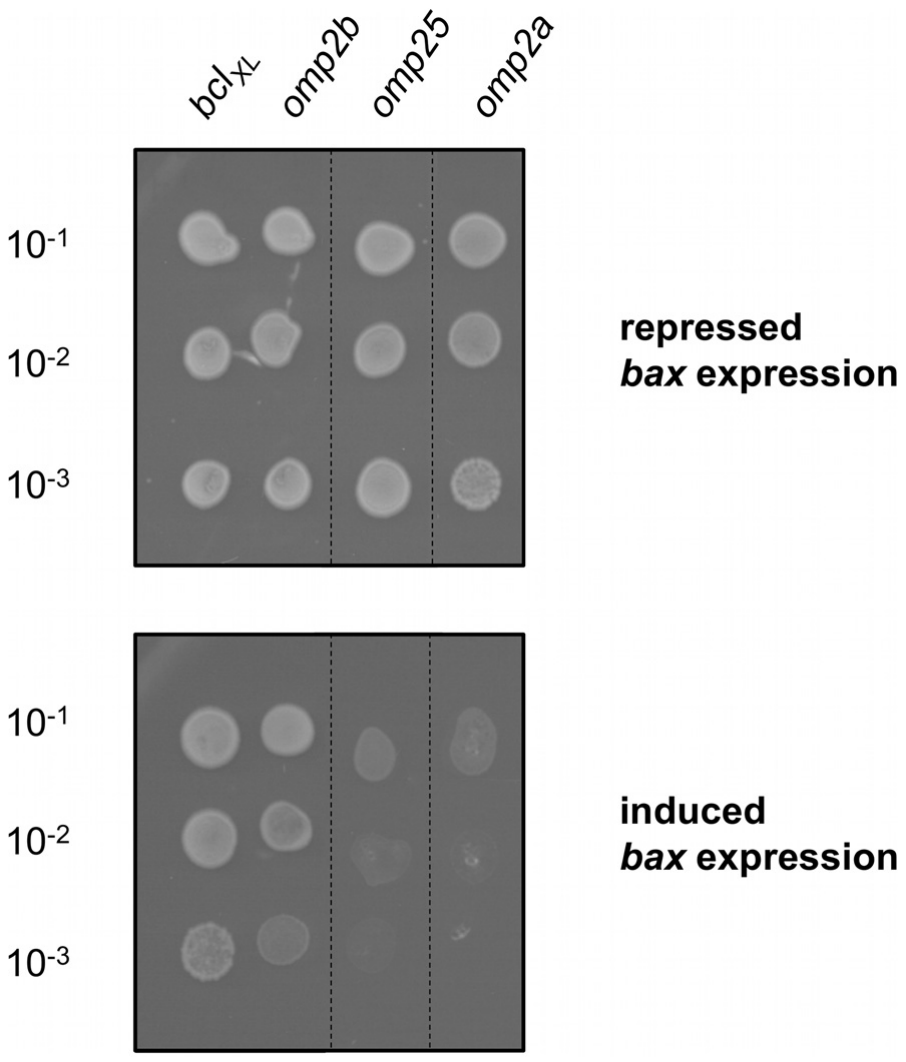
The Bax-suppressor effect of Omp2b is not common to *B. melitensis* outer membrane proteins. Drops of serial dilutions of saturated cultures, on media allowing repression or induction of *bax* and ORF expression, for GL0008 (*omp25*) and GL0007 (*omp2a*). GL0001 (*bcl_XL_*) and GL0004 (*omp2b*) strains are positive controls for growth. Dilution factors are indicated. Pictures were taken after 4 days of growth at 30°C.

Only two ORFs encode porin homologs (Omp2a and Omp2b) in the *B. melitensis* genome. Omp2b is encoded on the opposite strand and next to BMEI1306 ORF encoding Omp2a. Omp2b shares 83% identity with Omp2a. To assess the specificity of Omp2b in the inhibition of Bax-induced cell death in yeast, we tested the effect of Omp2a, but also of Omp25, a major *B. melitensis* outer membrane proteins (OMP), on the growth of yeast expressing *bax* (GL0007 and GL0008, respectively). None of them (3 independent clones tested by strain) was able to rescue yeast from Bax-induced cell death (Figure 3). The production of those proteins in GL0007 and GL0008 has been verified by Western blot (Figure S1 of Supporting information), suggesting that their inability to rescue yeast from Bax-induced cell death is not due to the absence of the OMPs. Their expression had no effect on the growth of the wild type yeast strain BF264-15 Dau (GL0014 and GL0015, data not shown), arguing against their hypothetical toxicity for this strain. Thus, the “anti-Bax” effect of Omp2b is specific to this OMP, since it is not even observed for Omp2a although both porins share high sequence similarity. Omp2a predicted signal peptide is identical to the shortened PSP of short-Omp2b. To determine if the functional difference between Omp2a and Omp2b can be explained by the difference in signal peptides, we constructed a version of Omp2a, in which the predicted signal peptide sequence has been replaced by the full Omp2b PSP (long-Omp2a). When expressed in yeast (GL0017), long-Omp2a was not able to prevent Bax-induced cell death, suggesting that although Omp2b PSP is required for Omp2b effect in yeast, other features in the porin are necessary and not shared by Omp2a (Figure S2 of Supporting information). *B. melitensis* Omp2a was shown to form a pore that is more efficient in sugar diffusion than Omp2b [25]. Whether the pore features are necessary for the function leading to the rescuing effect is presently unknown. Characteristic porin structures are composed of beta-barrels, with short periplasmic turns and external loops of variable length [33]. Interestingly, the external L3 and L5 loops are enriched in major structural differences between Omp2a and Omp2b [25], as between PorB porins from *Neisseria meningitidis* and *gonorrheae*, which have been characterized as showing opposite effects regarding host apoptosis [26], [27], [28].

Among other hypotheses on Omp2b mechanism of action in yeast, it would be tempting to speculate that the porin, once processed, might associate with mitochondria in yeast, given (i) the involvement of this organelle in Bax-induced lethality, (ii) the mitochondrial localization of neisserial PorB in infected cells [26], [27], [34], (iii) that ectopically produced bacterial β-barrels lacking their SP have been shown to locate to yeast mitochondria, by an import pathway shared with mitochondrial beta-barrels, involving import receptors and the TOB/SAM complex [35]. Alternatively, it is conceivable that Omp2b, in the cytosol, might prevent Bax mitochondrial targeting, either indirectly or by interacting with Bax directly. A pull-down experiment to test if Bax and Omp2b physically interact in yeast could not be performed since Omp2b is found in the insoluble fraction of lyzed yeast cells, which is not surprising considering its predicted tridimensional structure exposing hydrophobic side chains [25]. Nevertheless, it is completely conceivable that Omp2b prevents yeast cell death indirectly, that is without a physical interaction with Bax, taking into account the pleiotropic effects of Bax that have been proposed to be involved in yeast cell death [11].

This study is based on a screening method involving a functional assay in a "synthetic" system. Although several proteins previously identified by this strategy have been demonstrated to be involved in cell death inhibition in superior eukaryotic systems [14], [15], [16], [17], [18], Omp2b and FecE should be characterized further in a model that is more relevant to the natural infection conditions, such as transfection of constructs carrying these coding sequences in mammalian cells. Determining the role of Omp2b during infection should also rely on a reverse genetics approach aiming at the comparison of wild type and *omp2b* deletion strains of *B. melitensis* in their ability to inhibit apoptosis of infected cells. However, this was recluded by the essentiality of *omp2b* gene in *B. melitensis* (data not shown). The observation that Omp2a production in yeast does not induce a rescuing effect like Omp2b suggests that these differences between Omp2a and Omp2b might be related not only to porin functions but also to differences in apoptosis modulation.

The possible effect of Omp2b inside infected cells might be dependent on Omp2b delivery by outer membrane vesicles (OMV) that have been shown to harbor Omp2b [36] and serve as a secretion mechanism for effectors of other bacteria [37], or by the release of outer membrane fragments. It was indeed reported that *Brucella* LPS may travel to the host cell surface, supporting trafficking involving outer membrane components in infected cells [38], [39].

To the best of our knowledge, this study illustrates the first application of a screening strategy for the identification of Bax inhibitors in yeast to a bacterial genome. The proteins identified are anti-apoptotic effector candidates, of which the possible modulator effect on apoptosis can be characterized in the future in various mammalian cellular models of apoptosis.

## Materials and Methods

Features and references for the yeast and bacterial strains used in this study are available in Table 1. Primers sequences are available in Table S1 of Supporting information section. The construction and screening of the “*bax*+ORFeome” yeast library is described in details in the Supporting information section.

### Plasmids

pYES-DEST52-BMEI1305 (*omp2b*), pYES-DEST52-BMEI0660 (*fecE*), pYES-DEST52-BMEI1306 (*omp2a*), pYES-DEST52-BMEI1249 (*omp25*) and pYES-DEST52-*bcl_XL_* were obtained after LR recombination reaction (Gateway, Invitrogen) between pYES-DEST52 destination vector (Invitrogen 12286-019) and each corresponding pDONR201-ORF from the *B. melitensis* 16M ORFeome [23] or pDONR201-*bcl_XL_*. pDONR201-*bcl_XL_* was obtained after a BP recombination reaction (Gateway, Invitrogen) between pDONR201 and an amplification product of human *bcl_XL_* cDNA lacking the stop codon (from pcDNA3myc-*bcl_XL_*, kindly provided by T. Arnould, University of Namur, Belgium) flanked by *attB1* and *attB2* recombination sites (primers attB1-*bcl_XL_* and attB2-*bcl_XL_*). *t-omp2b* and *short-omp2b* were amplified from pYES-DEST52-*omp2b* with *t-omp2b*-attB1 and *t-omp2b*-attB2 primers, and from pYES-DEST52-*omp2b* with FWD*shortomp2b* and *t-omp2b*-attB2 primers, respectively. Each amplification product was cloned in pDONR201 by BP reaction. Resulting entry vectors were LR recombined with pYES-DEST52. Construction of pYES-DEST52-*long-omp2a* was based on a 2-steps PCR strategy. Fragment A was amplified from pYES-DEST52-*omp2b* with primers *long-omp2a*_1 and *long-omp2a*_2. Fragment B was amplified from pYES-DEST52-*omp2a* with primers *long-omp2a*_3 and *long-omp2a*_4. *long-omp2a* was amplified by overlap extension PCR with fragment A and B as matrixes, and with primers *long-omp2a*_1 and *long-omp2a*_4. Amplification product and pYES-DEST52-*omp2a* were cleaved by *Spe*I and *Asp*718I and then ligated to give pYES-DEST52-*long-omp2a*.

### Total protein extracts from *S. cerevisiae*

Extraction protocol was modified from [40] with 4% SDS in the SDS sample buffer.

### Antibodies used in Western blots

Bax was detected using HRP-coupled anti-Bax antibody (Santa Cruz sc-493 HRP), diluted 1∶100 in PBS-1% non-fat milk. Phosphoglycerate kinase was detected using mouse monoclonal anti-PGK antibody (Invitrogen Cat. No. A-6457), diluted 1∶10,000 in PBS-1% non-fat milk. His_6_-tagged fusions were detected using anti-6-His mouse monoclonal antibody (Covance Cat. No. MMS-156R), diluted 1∶1,000 in PBS-1% non-fat milk. Omp2b and variants described in this study were detected using monoclonal A6304D11G01 mouse antibody [25] as primary antibody. Tubulin was detected using mouse monocolonal anti-alpha-tubulin antibody (Sigma-Aldrich Cat. No. T5168) diluted 1∶50,000 in PBS-1% non-fat milk. Anti-mouse IgG, HRP-linked whole antibody (Amersham Cat. No. NA931, 1∶5000 diluted in PBS-1% non-fat milk) was always used as secondary antibody except for Bax detection.

## Supporting Information

Figure S1 Western blot showing that Omp2a or Omp25 is produced in BF264-15Dau yeast clones transformed with pYEp51-bax and pYES-DEST52-omp2a (GL0007) or pYES-DEST52-omp25 (GL0008). Three clones were tested for each strain. The detection was made with a monoclonal anti-His_6_ since Omp2a and Omp25 coding sequences are fused with a His_6_ tag in the pYES-DEST52 plasmids. The expected sizes of Omp2a and Omp25 fused to His_6_ are 43.9 and 27.6 kDa, respectively. Bax was detected with an anti-Bax antibody. The expected size of Bax is 21.2 kDa.
(0.12 MB PDF)

Figure S2 Replacing Omp2a signal peptide by Omp2b signal peptide does not confer to Omp2a the ability to save yeast from Bax-induced lethal effect. A fusion (long-omp2a) was constructed by fusing the coding sequence corresponding to Omp2b predicted signal peptide (PSP in main text) to the coding sequence corresponding to mature Omp2a (i.e. Omp2a without its signal peptide). (A) The expression of long-omp2a (GL0017) does not allow growth on a medium inducing Bax production. The clones expressing omp2b (GL0004) or omp2a (GL0007) are given as positive and negative controls for growth on this medium, respectively. (B) Western blot showing that long-Omp2a is detectable in yeast (strain GL0018) using the A63-04D11-G01 monoclonal antibody.
(0.40 MB PDF)

Table S1 Primer sequences.
(0.04 MB PDF)

Text S1 Supporting information regarding the Bax-rescue screening.
(0.08 MB PDF)
