# Supplementary figures and images for: Identification of the Essential Brucella melitensis Porin Omp2b as a Suppressor of Bax-Induced Cell Death in Yeast in a Genome-Wide Screening
Source: PLoS One. 2010 Oct 11;5(10):e13274. doi: 10.1371/journal.pone.0013274 (PMC2952587; doi:10.1371/journal.pone.0013274)

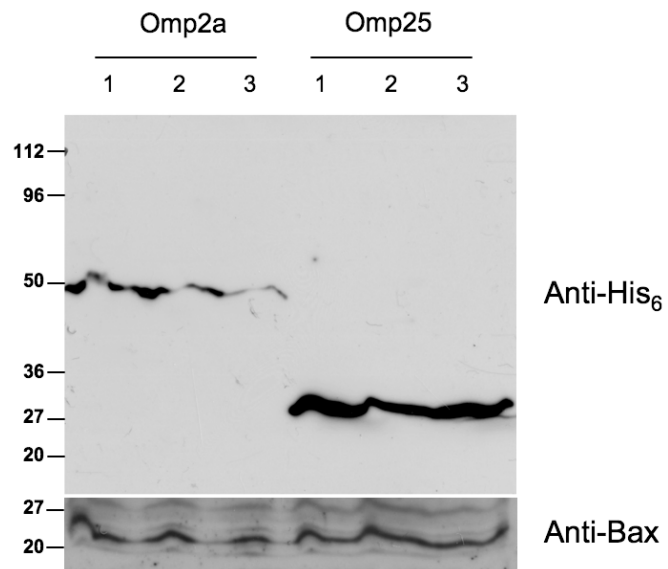

Supplement: Figure S1 — Western blot showing that Omp2a or Omp25 is produced in BF264-15Dau yeast clones transformed with pYEp51-bax and pYES-DEST52-omp2a (GL0007) or pYES-DEST52-omp25 (GL0008). Three clones were tested for each strain. The detection was made with a monoclonal anti-His6 since Omp2a and Omp25 coding sequences are fused with a His6 tag in the pYES-DEST52 plasmids. The expected sizes of Omp2a and Omp25 fused to His6 are 43.9 and 27.6 kDa, respectively. Bax was detected with an anti-Bax antibody. The expected size of Bax is 21.2 kDa. (0.12 MB PDF) [file pone.0013274.s001.pdf]

**A**

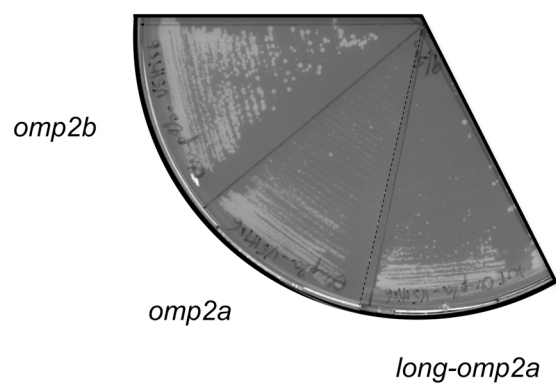

**B**

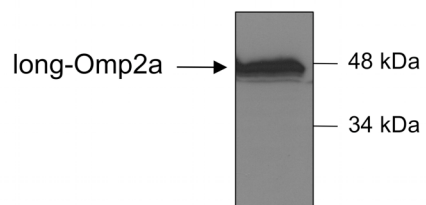

Supplement: Figure S2 — Replacing Omp2a signal peptide by Omp2b signal peptide does not confer to Omp2a the ability to save yeast from Bax-induced lethal effect. A fusion (long-omp2a) was constructed by fusing the coding sequence corresponding to Omp2b predicted signal peptide (PSP in main text) to the coding sequence corresponding to mature Omp2a (i.e. Omp2a without its signal peptide). (A) The expression of long-omp2a (GL0017) does not allow growth on a medium inducing Bax production. The clones expressing omp2b (GL0004) or omp2a (GL0007) are given as positive and negative controls for growth on this medium, respectively. (B) Western blot showing that long-Omp2a is detectable in yeast (strain GL0018) using the A63-04D11-G01 monoclonal antibody. (0.40 MB PDF) [file pone.0013274.s002.pdf]
