# Supplementary material for: Identification of the Essential Brucella melitensis Porin Omp2b as a Suppressor of Bax-Induced Cell Death in Yeast in a Genome-Wide Screening
Source: PLoS One. 2010 Oct 11;5(10):e13274. doi: 10.1371/journal.pone.0013274 (PMC2952587; doi:10.1371/journal.pone.0013274)
Supplement: Table S1 — Primer sequences. (0.04 MB PDF) [file pone.0013274.s003.pdf]

<u>**Supporting information**</u>

Table S1. Primers used in this study

Primer name	5’-3’ Sequence
attB1-*bcl*$_{XL}$	GGGGACAAGTTTGTACAAAAAAGCAGGCTATGGTACAGATACTCATCTCT
attB2-*bcl*$_{XL}$	GGGGACCACTTTGTACAAGAAAGCTGGGTTTTTCCGACTGAAGAGTGAGC
t-omp2b-attB1	GGGGACAAGTTTGTACAAAAAAGCAGGCTCGATGGACGCAATCGTCGCG
t-omp2b-attB2	GGGGACCACTTTGTACAAGAAAGCTGGGTCGAACGAACGCTGGAAGCG
FWDshort*omp2b*	GGGGACAAGTTTGTACAAAAAAGCAGGCTCGATGAACATCAAGAGCCTTCTC
pYES-DEST52_F	TAATACGACTCACTATAGGGA
pYES-DEST52_R	TCGAACCACTTTGTACAAGAA
