# Supplementary material for: Identification of the Essential Brucella melitensis Porin Omp2b as a Suppressor of Bax-Induced Cell Death in Yeast in a Genome-Wide Screening
Source: PLoS One. 2010 Oct 11;5(10):e13274. doi: 10.1371/journal.pone.0013274 (PMC2952587; doi:10.1371/journal.pone.0013274)
Supplement: Text S1 — Supporting information regarding the Bax-rescue screening. (0.08 MB PDF) [file pone.0013274.s004.pdf]

Supporting information

Text S1

Construction of the "*bax* + ORFeome" yeast library and screening for Bax inhibitors

All pDONR-201-ORFs of the *B. melitensis* ORFeome were used in 71 pools (each pool being the content of a half-plate of the ORFeome) in a LR recombination reaction with the pYES-DEST52 vector (Invitrogen 12286-019). The 71 pools of LR products were recovered after transformation of One Shot OMNIMAX 2T1^R *E. coli* (Invitrogen C8540-03), with the NucleoSpin® Multi 96 Plus Plasmid Kit (Macherey-Nagel 740625.24) and pooled in a superpool used to transform the *S. cerevisiae* QX95001 strain. Approximately 8800 clones were obtained on Sc-L-U (Sc medium lacking leucine and uracil) + 2 % glucose [1], and recovered as the "*bax* + ORFeome" yeast library. High efficiency yeast transformation was performed as described [2]. 2.6 x 10^5 CFU of this library were plated on Sc-L-U + 2% galactose and 1% raffinose (*pGAL1/10* induction). The numbers of clones obtained at each step of the library construction suggested a statistically complete coverage of the ORFeome diversity. After 3 days, 520 growing colonies (later divided in two categories regarding the relative sizes of colonies) were picked on new *pGAL1/10* induction plates (screening plates), and initial plates were submitted to cleaning. After 2 days, 80 more clones were picked from the cleaned plates on new screening plates. All screening plates were submitted to replica plating for cleaning and storage on Sc-L-U + 2% glucose (*pGAL1/10* repression). To test if the growing phenotype on *pGAL1/10* induction medium was plasmid-borne, candidates were picked from the screening plates on Sc-L + 2% glucose plates, grown for 2 days, then picked on Sc-L + 2% glucose + 5-fluoroorotate (5FOA) 0.1% plates to allow the selection of clones that lost the pYES-DEST52-ORF plasmid. Growing clones were picked on four types of media: *pGAL1/10* repression Sc-L plates with or without 0.15% 5FOA and *pGAL1/10* induction Sc-L plates with or without 0.15% 5FOA. Candidates considered to show a plasmid-borne growth phenotype are those able to grow on *pGAL1/10* repression Sc-L plates with or without 0.15% 5FOA, but not on *pGAL1/10* induction Sc-L plates with or without

0.15% 5FOA. A more stringent second run of screening for clones harboring a plasmid-borne phenotype was performed starting from storage plates. Two successive replicas were made on *pGAL1/10* repression plates with 0.25% 5FOA, at two days of interval, then plates were replicated on *pGAL1/10* induction Sc-L plates. 83.3% of the clones with a plasmid-borne phenotype in the second run of the screen were already selected in the first run. For the following identification steps, both candidates from the first and the second run were taken into account, except for candidates of the “small colony size” category for which only those that were selected in both runs were considered. 136 selected candidates were submitted to PCR with pYES-DEST52_F and pYES-DEST52_R primers. Amplification products were purified (MSB Spin PCRapace, Invitek) and sequenced with pYES-DEST52_F primer. 116 different ORFs were identified. The corresponding pDONR201-ORFs were individually picked in the ORFeome and used in 116 individual LR reactions with pYES-DEST52. Recombination products were recovered and used to individually transform *S. cerevisiae* QX95001 strain. Each co-transformed strain was streaked on a *pGAL1/10* repression Sc-L-U plate and a *pGAL1/10* induction Sc-L-U plate. For dilution drops, liquid cultures of each co-transformant were grown overnight; pellets were washed, resuspended, diluted in water (10X, 100X and 1000X) and spotted on *pGAL1/10* repression or induction Sc-L-U plates. Growth was observed after 4 days of incubation at 30°C.

[1] Walhout, A.J. and Vidal, M. (2001). High-throughput yeast two-hybrid assays for large-scale protein interaction mapping. Methods 24, 297-306.

[2] Agatep, R., Kirkpatrick, R.D., Parchaliuk, D.L., Woods, R.A. and Gietz, R.D. (1998). Transformation of Saccharomyces cerevisiae by the lithium acetate/single-stranded carrier DNA/polyethylene glycol (LiAc/ss-DNA/PEG) protocol. . Technical Tips Online 3, 133-137.
